# Gut Dysbiosis and Adult Atopic Dermatitis: A Systematic Review

**DOI:** 10.3390/jcm14010019

**Published:** 2024-12-24

**Authors:** Kevin Díez-Madueño, Pablo de la Cueva Dobao, Isabel Torres-Rojas, Marta Fernández-Gosende, Claudio Hidalgo-Cantabrana, Pablo Coto-Segura

**Affiliations:** 1 Dermatology Department, Hospital Universitario Infanta Leonor, Complutense University of Madrid, 28040 Madrid, Spain; kevin3diez@gmail.com; 2 School of Medicine, Complutense University of Madrid, 28040 Madrid, Spain; 3 Allergy Department, Hospital Universitario Infanta Sofía, 28702 Alcobendas, Spain; isatr93@hotmail.com; 4 MicroViable Therapeutics, 33006 Gijón, Spain; marta.gosende@microviable.com (M.F.-G.); claudio.hidalgo@microviable.com (C.H.-C.); 5 Dermatology Department, Hospital Vital Álvarez Buylla, 33611 Mieres, Spain; pablocotosegura@gmail.com

**Keywords:** atopic dermatitis, gut microbiota, intestinal dysbiosis, systematic review

## Abstract

**Background/Objectives:** Research on the relationship between gut microbiota (GM) and atopic dermatitis (AD) has seen a growing interest in recent years. The aim of this systematic review was to determine whether differences exist between the GM of adults with AD and that of healthy adults (gut dysbiosis). **Methods:** We conducted a systematic review based on the PRISMA guidelines (Preferred Reporting Items for Systematic Reviews and Meta-Analyses). The search was performed using PubMed, EMBASE, and Web of Science. Observational and interventional studies were analyzed. **Results:** Although the studies showed heterogeneous results, some distinguishing characteristics were found in the intestinal microbial composition of adults with dermatitis. Even though no significant differences in diversity were found between healthy and affected adults, certain microorganisms, such as Bacteroidales, Enterobacteriaceae, and *Clostridium* (*perfringens*), were more characteristic of the fecal microbiota in adults with AD. Healthy individuals exhibited lower abundances of aerobic bacteria and higher abundances of short-chain fatty acid-producing species and polyamines. Clinical trials showed that the consumption of probiotics (*Bifidobacterium* and/or *Lactobacillus*), fecal microbiota transplants, and balneotherapy modified the fecal microbiota composition of participants and were associated with significant improvements in disease management. **Conclusions:** In anticipation of forthcoming clinical trials, it is essential to conduct meta-analyses that comprehensively evaluate the effectiveness and safety of interventions designed to modify intestinal flora in the context of AD. Preliminary evidence suggests that certain interventions may enhance adult AD management.

## 1. Introduction

In recent years, interest in the role of gut dysbiosis (alterations in the fecal microbiota) and its association with various diseases, such as inflammatory bowel disease [1], autism spectrum disorder [2], cancer [3], and psoriasis [4] has grown exponentially. The microbiota is defined as the collection of bacteria, archaea, fungi, protozoa, and viruses that inhabit various locations of a multicellular organism [5]. Studies have previously demonstrated cutaneous dysbiosis on AD patients [6]. The GM is the most complex microbial consortium in humans, containing 10 times more cells than the host itself and representing over 1000 species [7]. The gut microbiome (the gene pool associated with a given microbiota) is 150 times richer than the human genome, ref. [8] providing a genetic reservoir capable of performing functions not encoded by human genes.

AD is the most common inflammatory skin disease in Western countries. Globally, AD affects approximately 20% of children and up to 10% of adults, with 4% of cases classified as severe [9]. Its prevalence has shown a concerning increase in recent years [10]. AD also significantly impacts the quality of life and psychosocial well-being of patients and their caregivers [11]. It is a common reason for consultations in primary and specialized care. Additionally, the chronic use of systemic treatments, short hospital stays due to poor disease control or complications, and its role as a relatively frequent cause of work absenteeism contribute to substantial healthcare costs. AD is a complex inflammatory dermatosis, and various relevant factors have been described for its pathophysiology: immune dysregulation (Th2-driven proinflammation, elevated IL-4, IL-5, IL-13, Ig E, eosinophilia), skin barrier disruption (reduced ceramides), skin dysbiosis (Staphylococcus aureus), intestinal dysbiosis (heterogeneous), genetic factors, and external factors. The disruption of the skin barrier is responsible for chronic inflammation with epidermal hyperplasia and cellular infiltrates (dendritic cells, eosinophils, and T cells). In particular, the overexpression of T cells leads to the release of chemokines and pro-inflammatory cytokines that promote IgE production, as well as local and systemic inflammation [12]. Currently, it is assumed that Th-2 and Th-22 lymphocytic populations play a common role in the development of inflammation and, consequently, epidermal hyperplasia in all AD subtypes. Th-2 (IL-4, IL-5, IL-13, IL-31) and Th-22 (IL-22) populations contribute to the inflammatory process that leads to epidermal proliferation in certain phenotypes [13,14]. At the same time, different ethnic profiles of AD and chronic forms exhibit distinct immune profiles, with a predominance of Th-1, Th-17, and others, leading to overexpression of various cytokines involved in AD (IL-5, IL-17, IL-12/23, IL-22, IL-9, IL-18, IL-33, IL-25, IL-37) and the release of other immunomodulatory molecules such as thymic stromal lymphopoietin (TSLP) [13,15,16,17].

While barrier function and genetic inheritance (intrinsic factors) are important, microbiome and environmental factors (extrinsic factors) appear to play a decisive role, as they contribute to the perpetuation of skin barrier dysfunction through a cyclical mechanism: increased itching, scratching, deterioration of permeability and filaggrin expression, and greater susceptibility to environmental aggression. Therefore, external or environmental biological factors have been studied as crucial in the development of eczema. The most influential factors in AD include ultraviolet light, demographic factors (urbanization), pollution and climate change, allergens, and skin and fecal microbiota [18]. One of the key determinants within the exposome concept and AD is the microbiome. The existence of a gut–skin axis is an evolving concept that has gained relevance in recent years. Additionally, the study of microbiomes is part of the emerging approach to studying inflammatory diseases from a multi-omic perspective (precision medicine), which is crucial for understanding AD.

The advent of next-generation sequencing technologies and bioinformatics has significantly advanced our understanding of GM. Massive sequencing and metagenomics (the study of microbiomes) have allowed for a more detailed exploration of host-immune system-GM interactions. The “gut-immunity-skin axis” may hold the key to understanding the increased incidence of immune-mediated diseases in Western countries. The microbiota can modulate both innate and adaptive immune responses, ref. [19] regulate gene expression, and influence epidermal proliferation and differentiation [20]. Gut dysbiosis during early life stages may trigger imbalances in immune tolerance and hinder immune maturation [21], potentially contributing to the development of the atopic march. Although the number of publications linking intestinal dysbiosis and AD continues to rise, the results remain controversial, and most studies focus on pediatric populations [22,23].

This review aims to compile and analyze the literature on GM in adults with AD, exploring the potential influence of the gut–skin axis on the disease. The objective was to review the existing literature on the GM of adults with AD and determine whether there are differences in the fecal microbiota between atopic and healthy adults (gut dysbiosis). Clinical trials on GM interventions for atopic patients were also analyzed to assess their efficacy and safety. Previous literature reviews have addressed this field of dermatology. Other systematic reviews have also been conducted; however, they encompass both pediatric and adult populations, leading to heterogeneity, include outdated techniques, and, ultimately, are less current.

## 2. Materials and Methods

We conducted a systematic review (January 2023) based on the PRISMA 2020 guidelines (Preferred Reporting Items for Systematic Reviews and Meta-Analyses) [24,25]. The search was performed using PubMed, EMBASE, and Web of Science. The following search algorithm was applied: (“dermatitis, atopic” AND “Gastrointestinal Microbiome”) OR ((“atopic dermatitis” OR “atopic eczema” OR “eczema”) AND (“fecal microbiota” OR “fecal microbiome” OR “gut microbiota” OR “gut microbiome” OR “intestinal microbiota” OR “intestinal microbiome”)).

**Figure 1:**
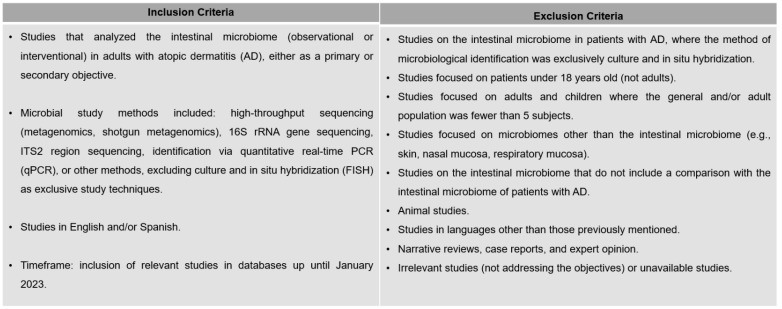
Eligibility Criteria for the systematic review. The reason for exclusion was multifactorial, with more than one reason contributing to the removal of each record. The reasons for exclusion included: background or context (n = 622), incorrect population (n = 564), incorrect publication type (n = 149), inadequate study design (n = 20), language (n = 3), and incorrect outcome variable (n = 2).

The review was conducted by two independent researchers (KDM and ITR). In the case of disagreement during article selection, a third supervising researcher (PCS) made the final decision (see Figure 1 and Supplementary Materials). To streamline the process of selecting and screening articles in systematic reviews, the authors used the Rayyan application. This work was conducted following a previous pilot study, in which different inclusion and exclusion criteria, as well as search strategies, were employed to maximize the number of studies retrieved while minimizing publication bias.

### 2.1. Eligibility Criteria

We included studies published in English or Spanish that analyzed the GM of human adults (>18 years) with AD using non-culture methods or in situ hybridization: quantitative PCR (qPCR) and/or next-generation sequencing (massive sequencing or 16S rRNA gene sequencing). Studies focused solely on pediatric populations, reviews, case reports, expert opinions, or other irrelevant articles were excluded (see Figure 1).

### 2.2. Data Extraction and Processing

Selected articles were categorized based on methodology as either observational or interventional studies (see Table 1, Table 2, Table 3 and Table 4). The results were analyzed by a multidisciplinary team.

## 3. Results

**Figure 2:**
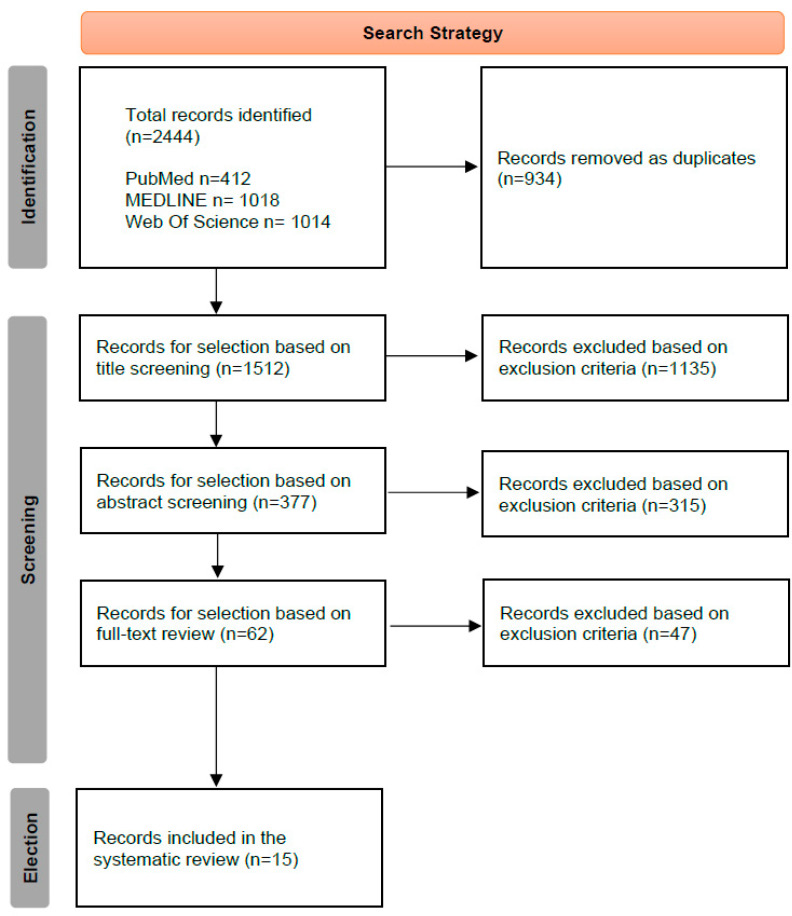
Search strategy.

The initial search yielded 2444 bibliographic records, of which 932 were excluded due to duplication. Of the remaining 1512 articles, 1450 were screened according to exclusion criteria, and 62 were selected for full-text review. Ultimately, 15 articles were included in the final analysis (six observational and nine interventional studies) [26,27,28,29,30,31,32,33,34,35,36,37,38,39,40]. Of these, 11 examined Eastern populations, while four focused on Western populations. Notably, 40% of the articles were published in 2022 (6/15), indicating a recent surge in interest in fecal microbiome studies related to atopy (Figure 2).

## 4. Discussion

AD is a chronic inflammatory, Th-2-dependent disease with a multifactorial origin, including genetic predisposition, epidermal barrier dysfunction, and environmental factors [41,42,43]. Previous research has demonstrated skin dysbiosis in atopic patients, characterized by reduced microbial diversity and an increased abundance of *Staphylococcus aureus.* In fact, this cutaneous dysbiosis has been associated with the development of flares and the perpetuation of the disease [6].

Gut dysbiosis can be defined as an imbalance found in the fecal microbiota associated with negative health implications [44]. Fecal dysbiosis disrupts gut composition and overall host homeostasis. This triggers an inflammatory response by increasing neutrophils, which not only act at the inflammation site but also affect surrounding tissues by releasing pro-inflammatory cytokines. The GM also produces ligands for pattern-recognition receptors (PRRs), which help protect the body and maintain a healthy microbial balance [45]. Recent studies suggest that neonatal gut microbiome alterations precede the development of atopy [46,47], and children with AD may exhibit gut dysbiosis [34,48]. This evidence suggests that inappropriate intestinal maturation (dysbiosis) in early life may lead to altered immune responses, predisposing individuals to atopy (allergies, asthma, and/or AD) in adulthood [49]. This compositional and functional alteration could lead to immune imbalance, a pro-inflammatory state, and the onset of disease [49]. Currently, most studies on GM aim to define the intestinal microbiome characteristic of a disease and determine the presence of associated dysbiosis by comparing the GM of a diseased group with that of a control group. The current definition of a healthy gut remains controversial. From an ecological perspective, when we talk about a “healthy gut”, we primarily refer to a microbiota characterized by stability. Stability is the combination of resistance (the ability of the microbial community to withstand changes) and resilience (the ability to return to equilibrium after disturbance) [50]. A more clearly defined concept is that of enterotypes. Enterotypes correspond to intestinal microbial patterns found in humans based on bacterial genera (composition) and conserved metabolic pathways (function). The existence of enterotypes suggests the presence of specialized ecological niches generating energy from available fermentable substrates through well-defined metabolic pathways. Enterotypes are stable, limited in number, and reflect the homeostasis between different bacterial communities. The definition of these enterotypes could, in the future, be associated with health and disease states and may lead to the unraveling of innovative endotypes and perspectives for several conditions [51].

Globally, in this review, no significant differences were observed in the composition of the GM in adult patients with AD compared to the control population, apart from some bacterial genera and families (see Table 2 and Table 4). Although studies indicate that children with AD exhibit reduced microbial diversity [52,53,54], this review did not find significant evidence of reduced fecal diversity in adults with dermatitis. Overall, we did not find global differences in the GM between adults with dermatitis and healthy adults. However, certain bacterial species and genera, as well as some fecal conditions, were more characteristic of the diseased group, while others were more characteristic of the healthy group:

- Bacterial genera more commonly found in adults with AD (“pathogenicity-associated bacteria”) included the order Bacteroidales (genus *Bacteroides*) [29,31], the family Enterobacteriaceae (genera *Escherichia*-*Shigella*) [30,34], and *Clostridium* (*perfringens*) [38] (Table 2 and Table 4).
- Bacterial genera more abundant in healthy adults (“protection-associated bacteria”) included *Prevotella* [31], *Lactobacillus*, *Streptococcus*, *Bifidobacterium*, *Clostridium* [34] (*Clostridium* cluster IV and subcluster XIV) [39], and *Faecalibacterium* (*prausnitzii*) [33] (see Table 2 and Table 4).

In general, the GM of the healthy population presented a greater abundance of species typically producing short-chain fatty acids (SCFAs) and polyamines, a lower abundance of aerobic microorganisms, and conserved microbial diversity.

Although the definitions of dysbiosis and a “healthy gut” are still unclear, some signatures of dysbiosis in AD have been described:

- An elevated Firmicutes/Bacteroidetes ratio has been associated with AD [32,37].
- Children with AD present lower intestinal diversity compared to healthy children [52,53,54]. However, this has yet to be demonstrated in adults with dermatitis.
- Patients with dermatitis tend to present a reduced abundance of SCFA-producing bacterial genera (*Bifidobacterium*, *Lactobacillus*, *Clostridium*, *Bacteroides*, or *Streptococcus*), with a lower capacity to induce immunotolerance (lower induction of regulatory T cells [Treg]) [34].

The GM could play a significant role in the activation of dendritic cells and the expression of regulatory T cells (Th1, Th2, Th17, or Treg), actively participating in the concepts of immunotolerance and immunoregulation [55,56,57]. The concept linking the skin, immune system, and GM is known as the “gut-skin axis”. The relationship between the gut microbiome and the development of AD is complex, multifactorial, and remains under investigation. Among the various mechanisms underlying the gut–skin relationship and the development of dermatitis, the following have been identified:

- Gut dysbiosis and the microbiota–intestinal epithelium interaction (leaky gut theory) [48].
- Environmental-dependent intestinal dysbiosis (environmental pathway and hygiene hypothesis) [58,59].
- Gut maturation and immune system development and modulation (microbiota-immune system pathway) [46,60].
- Intestinal dysbiosis, metabolic alterations in the fecal environment, and immunomodulation (SCFA metabolism, tryptophan pathway, and other mechanisms under investigation) [61,62].
- Intestinal dysbiosis, microbiota, and genetic expression (genetic, epigenetic, and cellular modulation pathways [20,63].

Some authors suggest that patients with AD may exhibit dysfunction in the intestinal barrier (“leaky gut”) and gut dysbiosis, characterized by a flora with reduced production of SCFAs. A permeable intestinal barrier could allow an increased penetration of toxins and pathogens systemically, leading to the release of pro-inflammatory cytokines (IL-25, IL-33, and TSLP), which would drive monocyte migration and differentiation into macrophages, as well as an increased differentiation of T cells into Th2 cells. Additionally, in patients with AD, an imbalance between IgA and IgE is found in the intestinal lumen, with elevated IgE levels and mast cells infiltrating the intestinal lamina propria, causing an inflammatory response [64].

Gut dysbiosis would, in turn, lead to an enhanced Th2-dependent response through alterations in microbial composition (pathogenic microorganisms) and function (metabolism of SCFAs, tryptophan, polyamines, and other nutrients). These alterations in composition and function provide a basis for greater colonization by pathogenic microorganisms and a reduced presence of butyrate- and propionate-producing bacteria, perpetuating a cycle of inflammation and dysbiosis [48,64].

SCFAs are products of carbohydrate fermentation by fecal bacteria. The primary SCFAs are acetate, propionate, and butyrate. As previously noted, subjects with AD may present with gut dysbiosis in early life. This dysbiosis leads to impaired intestinal maturation, with reduced fecal diversity and dysfunction in SCFA metabolism and other metabolites [52]^.^ In AD, an imbalance between commensal populations (*Lactobacillus* and *Bifidobacterium*) and pathogenic species results in lower SCFA production and an increased inflammatory response. Various studies confirm that lower butyrate [63] and propionate [62] levels are associated with AD. An adult reaches a physiological GM through progressive maturation, in which facultative aerobic and anaerobic species (*Streptococcus*, *Staphylococcus*, or *Enterobacteriaceae*) are gradually displaced by strict anaerobes that produce SCFAs (*Bifidobacterium*, *Bacteroides*, or *Clostridium*) [54].

SCFAs confer protection against atopy through various mechanisms:

- They create an environment favorable for the dominance of anaerobic microbial species (a “healthier gut-like” environment), reducing the abundance of pathogenic species and breaking the dysbiosis-inflammation cycle [61].
- Butyrate is an activator of PPAR-γ (peroxisome proliferator-activated receptor gamma), mitochondrial respiration, and oxygen consumption via oxidative phosphorylation. This preserves an anaerobic environment, reduces epithelial proliferation, and mitigates Th2-dependent responses [54].
- Butyrate induces Treg cells and promotes immunotolerance [65]. In AD patients, this cyclic interaction is diminished, with an increased inflammatory response [54].

The release of indole-3-carboxaldehyde (a product derived from tryptophan metabolism) stimulates the aryl hydrocarbon receptor (AHR), downregulating Th2 responses. Some *Bifidobacterium* species produce indoles that may lead to improvements in AD [32].

Polyamines (putrescine, spermidine, and spermine) are bacterial metabolic products. AD individuals have reduced fecal polyamine levels. This polyamine deficiency is associated with intestinal barrier dysfunction and an imbalance in cytokine and immunoglobulin release [26]. Certain probiotics (containing SCFA- and polyamine-producing strains) may reduce AD activity via a Th1 response [39].

Probiotics, also referred to as live biotherapeutic products, are defined by the FDA as products containing live organisms, such as bacteria, that naturally inhabit the human body. These are live microorganisms that, when administered in adequate amounts, confer health benefits to the host [66]. The most common probiotics are bacteria from the *Lactobacillus* and *Bifidobacterium* genera. The use of probiotics is based on their ability to induce changes in the composition and function (predominant metabolism) of a potentially dysbiotic or “unhealthy” environment. The use of probiotics to alleviate AD symptoms is justified by their potential to address underlying gut dysbiosis. Probiotics could induce indirect changes in the Th1/Th2 balance, helping to control AD symptoms [39].

Proposed mechanisms:

- Immunotolerance: SCFAs produced by probiotic bacteria can stimulate Treg responses, reducing serum levels of IL-4, IL-5, and TSLP [67].
- Microbial balance: probiotic strains may promote a favorable fecal environment for the colonization of health-associated microorganisms, preventing pathogen overgrowth and breaking the dysbiosis–inflammation cycle;
- Th2 downregulation: probiotics can reduce Th2 responses by inhibiting NF-κB (nuclear factor kappa B) and activating AHR [67].

In our review, clinical trials that used probiotics demonstrated significant statistical improvement in disease scales and symptoms (6/7). None reported adverse effects. Probiotic consumption showed transmission of the microbial strains to the patients’ GM (6/7) [33,34,35,36,37,38]. The efficacy of probiotics in managing AD has been investigated by other researchers. Kim et al. conducted a systematic review and meta-analysis evaluating the effectiveness of probiotics in AD treatment (in both children and adults), stratifying the results by geographic subgroups, disease severity, intervention duration, and probiotic strain. This meta-analysis concludes that there is scientific evidence supporting the supplementation with probiotics as potentially beneficial in the treatment of AD [68]. However, the findings of our review and those of other authors contrast with results reported by other studies. A Cochrane review published in 2018 assessed the therapeutic efficacy of probiotics in AD. This review analyzed 39 clinical trials (2599 participants), including both children and adults. The authors concluded that the probiotics available at that time lacked differential efficacy in improving symptoms, patient-perceived quality of life, or investigator-assessed disease severity [69].Given the discordance between studies, conducting further meta-analyses to evaluate this topic is warranted to derive more robust conclusions based on higher levels of evidence. Further trials with larger sample sizes, metagenomic sequencing, extended follow-ups, and different bacterial species are essential to determine the efficacy and safety of probiotics in adult AD.

The GM in adults and infants.

The GM of adults and infants differs compositionally, with the adult GM being more diverse and exhibiting less interpersonal variability [70]. Although the most significant impact on GM intervention occurs during infancy, adult GM retains some plasticity, and intervention could alter disease progression [28,71]. Since the most critical period for GM plasticity occurs within the first year of life, early probiotic use in infants at high risk of developing atopy could be valuable for prevention [72,73].

Risk factors for developing immune-allergic diseases include:

- Maternal factors: gut and vaginal dysbiosis, smoking, diet [74] and stress during pregnancy [75].
- Cesarean delivery: no exposure to vaginal flora [35].
- Formula feeding: replacing breastfeeding with artificial milk [76].
- Antibiotic use: during the perinatal period [77] and early childhood [78].
- Diets low in vegetables, fruits, and omega-3 fatty acids [79].
- Western culture and environment [77].
- Loss of contact with animals [80].

## 5. Recommendations

Our systematic review does not support making recommendations regarding the use of probiotics or other types of supplementations for the treatment or prevention of AD. A guideline promoted by the World Allergy Organization (WAO), suggests the use of prebiotic supplementation in infants who are not exclusively breastfed. This recommendation is conditional and is based on a very low certainty of evidence. Conversely, the organization decided not to issue recommendations regarding the use of prebiotics during pregnancy or breastfeeding for the prevention of AD, as no observational or experimental studies on this topic were identified [73]. A Cochrane review conducted in 2018 evaluated the therapeutic efficacy of probiotics as a treatment for AD. At that time, the study concluded that the available probiotics lacked differential efficacy in improving symptoms or the quality of life of patients [69]. The contradictions among studies highlight the need for further research involving larger sample sizes and the development of meta-analyses. Additionally, considering the pathophysiology of AD and various factors previously discussed regarding intestinal maturation, it is essential to conduct studies with longer-term prospective follow-up, particularly during early childhood and/or breastfeeding stages.

## 6. Study Limitations

Our review may be subject to various biases, including publication bias. To mitigate this, the review was conducted using three search engines and included articles in two languages. The number of articles analyzed was relatively small (n = 15), and a significant proportion of the studies involved Asian populations (73.3%). This limitation is important because differences in enterotypes between Asian and Western populations have been documented, and an “Asian” phenotype of AD has been described [81]. Despite strict eligibility criteria, the selected studies were heterogeneous in their objectives, methodology, and results. There were notable differences in the microbial analysis techniques used, with only two studies employing metagenomic analysis. Currently, understanding the functional gene expression of an ecosystem (metagenomics) is far more relevant than compositional data [82], and future research should focus on this area.

## 7. Conclusions

The role of the GM in the pathophysiology of AD represents a rapidly evolving, yet still-contentious, area of research. While the studies included in our review displayed heterogeneous results regarding the composition of the fecal microbiota, several key microbial signatures were noted. Although no significant differences in overall microbial diversity between healthy individuals and AD patients were consistently observed, certain taxa—such as *Bacteroidales*, *Enterobacteriaceae*, and *Clostridium perfringens*—were more commonly associated with AD. Conversely, healthy microbiota was typically enriched with anaerobic bacteria that produce SCFAs and polyamines, molecules known to promote gut health and modulate immune responses.

Despite the variation in methodologies across the studies, clinical trials highlighted promising therapeutic interventions. The use of probiotics, particularly those containing *Bifidobacterium* and *Lactobacillus* strains, along with fecal FMT, significantly altered gut microbial composition in participants and correlated with substantial improvements in disease control. These findings underscore the therapeutic potential of modulating the GM in AD, opening a novel avenue for disease management.

Future research, particularly studies utilizing advanced metagenomic sequencing techniques, is essential to deepen our understanding of these microbial communities and their functional roles. Standardization of therapeutic protocols, including probiotic use and FMT, is also critical to establish evidence-based clinical practices. The emerging interplay between the GM and AD may offer transformative insights and interventions for both prevention and treatment.

## Figures and Tables

**Table 1:** Analysis of observational studies.

Title	Authors	Journal	Year	Nationality	Design	Primary Objective	Comparison Groups	Sample	Microbial Analysis Method
Comparison of Fecal Microbiota and Polyamine Concentration in Adult Patients with Intractable Atopic Dermatitis and Healthy Adults	Matsumoto et al.	Allergol Immunopathol (Madr).	Y2007	Japan	Case-control study	To compare intestinal polyamine levels between atopic and healthy individuals, and to study fecal microbiota between atopic and healthy individuals using T-RFLP	Adults with atopic dermatitis vs. healthy adults	24 (11 AD vs. 13 healthy)	Terminal restriction fragment length polymorphism (T-RFLP) of the 16S rRNA gene
Allergy associations with the adult fecal microbiota: Analysis of the American Gut Project	Hua et al.	EBioMedicine	Y 2016	US	Retrospective descriptive study	To determine whether there is an association between the development of allergies and the presence of intestinal dysbiosis in adults.	Analysis of publicly available results from the American Gut Project (fecal samples) of adults with different types of allergies (food allergy, non-food allergy, eczema, asthma, hay fever)	1879 pacientes (318 AD vs. 1561 healthy)	16S rRNA gene sequencing
Inverse Association Between the Skin and Oral Microbiota in Atopic Dermatitis	Li et al.	J Invest Dermatol	Y 2019	China	Case-control study	To conduct a comparative analysis of the microbiota in the skin, oral cavity, and gut of patients with AD and to understand the relationships between the microbiota of these three habitats and the host with AD	Patients with atopic dermatitis vs. healthy patients	78 fecal samples (38 from adolescents and adults with atopic dermatitis vs. 38 from healthy adolescents and adults)	16S rRNA gene sequencing
Differences in gut microbiota between allergic rhinitis, atopic dermatitis, and skin urticaria	Su et al.	Medicine (Baltimore)	Y 2021	Taiwan (China)	Prospective Descriptive Study	To perform a comparative analysis of the microbiota in the skin, oral cavity, and gut of patients with atopic dermatitis, and to understand the relationships between the microbiota in these three habitats and the host with atopic dermatitis	Adults with atopic dermatitis, rhinitis, and urticaria	39 participants (19 with atopic dermatitis, 9 with urticaria, and 11 with allergic rhinitis)	16S rRNA gene sequencing
Study of The specificity of gut microbiota in adult patients with delayed-onset of atopic dermatitis	Liu et al.	Allergol Immunopathol (Madr).	Y2022	China	Case-control study	To compare the differences in gut microbiota between adults with late-onset atopic dermatitis, persistent atopic dermatitis, and healthy controls.	32 adults (12 with adult-onset atopic dermatitis, 10 with persistent atopic dermatitis, and 10 healthy controls)	32 participants (12 with adult-onset atopic dermatitis, 10 with persistent atopic dermatitis, and 10 healthy controls)	16S rRNA gene sequencing
Exploring the Differences in the Gut Microbiome in Atopic Dermatitis According to the Presence of Gastrointestinal Symptoms	Han et al.	J Clin Med	Y 2022	Korea	Case-control study	To describe the differences in composition, richness, and distinctive taxa of the gut microbiota between patients with atopic dermatitis with and without gastrointestinal symptoms	A cohort of patients with atopic dermatitis was divided based on gastrointestinal symptoms into: (1) AD with epigastric fullness (ADwEF), (2) AD with epigastric rigidity (ADwER), and (3) AD without gastrointestinal symptoms (ADw/oGI), and compared to healthy controls. We have four groups: AD without symptoms, AD with epigastric fullness, AD with epigastric rigidity, and non-AD controls.	27 participants (20 with atopic dermatitis [7 without gastrointestinal symptoms, 7 with epigastric fullness, and 6 with epigastric rigidity] vs. 7 healthy controls)	16S rRNA gene sequencing

**Table 2:** Summary of the most noteworthy and/or significant results from the compositional analysis of observational studies.

Observational Studies Selected and/or Notable Statistically Significant Results from the Composition Study	
Exploring the Differences in the Gut Microbiome in Atopic Dermatitis According to the Presence of Gastrointestinal Symptoms	The most abundant bacterial family in patients with AD was Bacteroidaceae, while the most abundant in healthy individuals was Prevotellaceae. In patients with AD (and gastrointestinal symptoms), symptoms increased when levels of *Prevotella copri* decreased and levels of Bacteroides increased. Patients with AD and gastrointestinal symptoms exhibit a distinct microbiota. The group with AD and symptoms of epigastric rigidity showed low diversity and uniformity. This group demonstrated greater abundance of *Bacteroides* and lower abundance of *Prevotella* compared to AD patients without gastrointestinal symptoms or healthy controls.
Study of The specificity of gut microbiota in adult patients with delayed-onset of atopic dermatitis	In the group of AD that debuted in adulthood, the predominant genus was *Escherichia*-*Shigella*. In this group, *Agathobacter* and *Dorea* were significantly reduced, while the relative level of the *Bacteroides pectinophilus* group increased notably compared to the other two groups. In the persistent AD group, *Faecalobacterium* was the predominant genus. In the healthy group, the predominant genus was *Subdoligranulum*.
Differences in gut microbiota between allergic rhinitis, atopic dermatitis, and skin urticaria	The microbiota in patients with skin allergies (AD and urticaria groups) differs significantly from that in patients with allergic rhinitis, suggesting the existence of distinguishable gut–skin and gut–nose axes. An increase in species from the phylum Firmicutes, species from the order Bacteroidales, and the family Ruminococcaceae (*Clostridia*) are confirmed as typical characteristics of intestinal dysbiosis in patients with allergic diseases.
Allergy associations with the adult fecal microbiota: Analysis of the American Gut Project	Adults with allergies, particularly to nuts and seasonal pollen, exhibit low diversity, a reduction in Clostridiales, and an increase in Bacteroidales in their microbiota (dysbiosis). The low richness and altered microbiota composition were not significantly associated with AD.
Inverse Association Between the Skin and Oral Microbiota in Atopic Dermatitis	The skin and oral cavity of patients with AD exhibited a differential reduction in microbial diversity, which correlated distinctly with the severity of the disease. This was not evidenced at the fecal level.
Comparison of Fecal Microbiota and Polyamine Concentration in Adult Patients with Intractable Atopic Dermatitis and Healthy Adults	Adults with AD exhibited reduced fecal levels of polyamines (putrescine and spermidine) compared to controls. Atopic individuals showed elevated levels of Enterobacteriaceae (bacteria that compromise the intestinal barrier through the absorption of polyamines). Low concentrations of polyamines may be associated with AD in adults.

**Table 3:** Analysis of interventional studies.

Title	Authors	Journal	Year	Nationality	Primary Objective	Microbial Analysis Method	Starting Sample	Final Sample	Gender	Age	Intervention	Design	Dosing	Follow-Up	Outcome	Safety
*Bifidobacterium longum* mediated tryptophan metabolism to improve atopic dermatitis via the gut-skin axis	Fang et al.	Gut Microbes	Year 2022	China	Exploring microbial metabolism and the influence of Bifidobacterium longum on DA.	16S rRNA gene sequencing	87 atopic dermatitis (44 probiotic vs. 43 placebo)	86 atopic dermatitis (43 probiotic vs. 43 placebo)	Female: 58 Male: 34	49	B. longum CCFM1029 (10^9^ CFU/2 g)	Randomized, placebo-controlled trial.	Daily for 8 weeks.	8 weeks	Significant reduction in SCORAD and DLQI.	No adverse effects reported
Changes in Gut Microbiota of Patients with Atopic Dermatitis During Balneotherapy	Thirion et al.	Clin Cosmet Investig Dermatol	Year 2022	France	To determine whether there is an improvement in the control of DA following balneotherapy, by measuring changes in microbial indicators using Shotgun metagenomics.	Shotgun metagenomics	96 patients with atopic dermatitis (48 with long-standing atopic dermatitis vs. 48 with short-term atopic dermatitis)	96 patients with atopic dermatitis (48 with long-standing atopic dermatitis vs. 48 with short-term atopic dermatitis)	Female: 51 Male: 45	40	La Roche-Posay thermal spring water (LRP-TSW)	Open-label trial.	Daily for 18 days	18 days	Significant reduction in SCORAD	No adverse effects reported
Effect of a Novel E3 Probiotics Formula on the Gut Microbiome in Atopic Dermatitis Patients: A Pilot Study	Wang	Biomedicines	Year 2022	China	To evaluate the clinical efficacy and the evolution of GM in patients with DA following the intervention of a probioti	16S rRNA gene sequencing	41 patients with atopic dermatitis (open-label trial)	41 patients with atopic dermatitis (open-label trial)	Female: 25 Male: 16	47	Probiotic E3 (a mixture of prebiotics [fructooligosaccharides, galactooligosaccharides, and inulin], probiotics [*Lactobacillus rhamnosus* GG, *Lactobacillus acidophilus* GKA7, *Lactococcus lactis* GKL2, *Lactobacillus casei* GKC1, *Lactobacillus paracasei* GKS6, *Bifidobacterium bifidum* GKB2, and *Bifidobacterium lactis* GKK2], and postbiotics [heat-killed L. plantarum (HK-LP)]).	Open-label trial.	Daily	8 weeks	Significant reduction in EASI	No adverse effects reported
Clinical efficacy of fecal microbial transplantation treatment in adults with moderate-to-severe atopic dermatitis	Mashiah	Immun Inflamm Dis.	Year 2022	Israel	To evaluate the transmission of bacterial strains, as well as the efficacy and safety of fecal microbiota transplantation in patients with DA	Shotgun metagenomics	15 patients with atopic dermatitis (cross-over trial).	9 patients with atopic dermatitis (cross-over trial).	Female: 4 male: 9	45	Fecal transplantation from 3 healthy donors.	Single-blind, crossover, placebo-controlled trial.	Biweekly. Two placebo interventions separated by 2 weeks, followed by four fecal transplant interventions, each separated by 2 weeks	18 weeks	Significant reduction in SCORAD	No adverse effects reported
Probiotics modulate the gut microbiota composition and immune responses in patients with atopic dermatitis: a pilot study	Fang et al.	Eur J Nutr	Year 2020	China	Determine the effects of probiotics on clinical outcomes, immune response, and metabolic inflammation in patients with atopic dermatitis	16S rRNA gene sequencing	109 patients with atopic dermatitis: placebo group (26), oligosaccharides group (11), Bifidobacterium bifidum CCFM16 group (29), and Lactobacillus plantarum CCFM8610 group (43)	104 patients with atopic dermatitis: placebo group (25), oligosaccharides group (10), Bifidobacterium bifidum CCFM16 group (28), and Lactobacillus plantarum CCFM8610 group (41)	Female: 67 Male: 42	52	Probiotic groups: *Bifidobacterium bifidum* CCFM16 and *Lactobacillus plantarum* CCFM8610. Daily dosage: 10^9^ CFU. Oligosaccharides group: Oligosa.	Randomized	Daily	8 weeks	Significant reduction in SCORAD	No adverse effects reported
Antipruritic effects of the probiotic strain LKM512 in adults with atopic dermatitis	Matsumoto et al.	Ann Allergy Asthma Immunol	Year 2014	Japan	Determine the effects of the probiotic Bifidobacterium animalis subsp. lactis LKM512 on metabolite expression and metabolic inflammation in patients with atopic dermatitis.	Quantitative real-time PCR for selected microorganisms; terminal restriction fragment length polymorphism (T-RFLP) of the 16S rRNA gene.	44 patients with atopic dermatitis (22 probiotic vs. 22 placebo)	participants	Female: 20 Male: 24	34	Probiotic: *Bifidobacterium animalis* subsp. *lactis* 6 × 10^9^ CFU. Placebo: excipient.	Randomized, double-blind, placebo-controlled clinical trial.	Not specified in the text	8 weeks	Significant reduction in pruritus and improvement in quality of life.	No adverse effects reported.
Effects of *Lactobacillus salivarius* LS01 (DSM 22775) treatment on adult atopic dermatitis: a randomized placebo-controlled study	Drago et al.	Int J Immunopathol Pharmacol	Year 2011	Italy	Determine the clinical efficacy of the intake of a probiotic strain (*Lactobacillus salivarius LS01*) in the treatment of adults with atopic dermatitis.	Quantitative PCR for selected microorganisms	38 patients with atopic dermatitis (19 probiotic vs. 19 placebo)	38 patients with atopic dermatitis (19 probiotic vs. 19 placebo)	Female: 20 Male: 18	30	Probiotic: *L. salivarius* LS01 1 × 10^9^ CFU. Placebo: maltodextrin	Double-blind, placebo-controlled trial.	Daily (twice/day)	16 weeks	Significant reduction in SCORAD and DLQI.	No adverse effects reported.
The effect of probiotics on faecal microbiota and genotoxic activity of faecal water in patients with atopic dermatitis: a randomized, placebo-controlled study	Roessler et al.	Clin Nutr	Year 2011	Germany	Determine whether the probiotic complex Lactobacillus paracasei Lpc-37, Lactobacillus acidophilus 74-2, and Bifidobacterium animalis subsp. lactis DGCC 420 can affect the microbiota and its genotoxic activity in healthy subjects and patients with atopic dermatitis.	Quantitative PCR for selected microorganisms	30 participants (15 with atopic dermatitis vs. 15 healthy controls).	30 participants (15 with atopic dermatitis vs. 15 healthy controls).	Female: 22 Male: 8	24	Probiotic: *Streptococcus thermophilus* enriched with probiotic strains *L. paracasei* Lpc-37 (3.9 × 10^8^ CFU/g), *L. acidophilus* 74-2 (2.9 × 10^4^ CFU/g), and *B. animalis* subsp. *lactis* DGCC 420 (B. lactis 420, 5.9 × 10^4^ CFU/g).	Double-blind, crossover, placebo-controlled trial.	Daily	20 weeks	None measeured	No adverse effects reported.
LKM512 yogurt consumption improves the intestinal environment and induces the T-helper type 1 cytokine in adult patients with intractable atopic dermatitis	Matsumoto et al.	Clinical Exp Allergy	Year 2007	japan	Determine the effect of probiotic yogurt (Bifidobacterium animalis subsp. lactis LKM512) on symptoms, Th1/Th2 response, metabolic inflammation, levels of polyamines, and short-chain fatty acids in patients with refractory atopic dermatitis.	Terminal restriction fragment length polymorphism (T-RFLP) 16S rRNA gene	10 patients with atopic dermatitis (cross-over trial)	10 patients with atopic dermatitis (cross-over trial)	Female: 6 Male: 4	22	Probiotic: fermented with B. animalis subsp. lactis LKM512 and Lactobacillus delbrueckii subsp. bulgaricus. Placebo: fermented with Streptococcus thermophilus LKM1742.	Double-blind, crossover, placebo-controlled trial.	Daly	8 weeks	Reduction of itching and burning. It is not determined whether the reduction is significant.	No adverse effects reported.

**Table 4:** Summary of notable and some statistically significant results from the compositional analysis of interventional studies.

Interventional Studies Selected and/or Notable Statistically Significant Results from the Composition Study	
*Bifidobacterium longum* mediated tryptophan metabolism to improve atopic dermatitis via the gut-skin axis	*B. longum* significantly remodeled the microbiota and enhanced the production of indole-3-carboxaldehyde through tryptophan metabolism.
Changes in Gut Microbiota of Patients with Atopic Dermatitis During Balneotherapy	During balneotherapy, significant changes were observed in microbiota composition and improvement in the disease. The bacteria *Lachnospira pectinoschiza* and *Faecalibacterium prausnitzii* (both belonging to the Class Clostridia) showed a positive correlation with SCORAD (protective against AD). Other species from the Clostridia and Actinobacteria classes correlated negatively.
Effect of a Novel E3 Probiotics Formula on the Gut Microbiome in Atopic Dermatitis Patients: A Pilot Study	Responders exhibited a higher abundance of protective bacteria, including *Clostridium*, *Erysipelatrichaceae*, *Faecalibacterium*, *Lactobacillus*, *Romboutsia*, and *Streptococcus*, alongside a reduced relative abundance of *Collinsella*, *Fusicatenibacter*, and *Escherichia*-*Shigella* (bacteria associated with atopic dermatitis). The species richness of responders was significantly greater following the intervention and was compositionally more similar to that of healthy subjects.
Clinical efficacy of fecal microbial transplantation treatment in adults with moderate-to-severe atopic dermatitis	Following the transplant, metagenomic analysis of the gut demonstrated the transmission of bacterial strains with greater similarity between donor and patient samples. Among the transmitted bacteria, *Lachnospiraceae* (*Clostridia*) and *Prevotella copri* (*Bacteroidia*) were particularly notable.
Probiotics modulate the gut microbiota composition and immune responses in patients with atopic dermatitis: a pilot study	The administration of *Lactobacillus plantarum* significantly influenced alpha diversity and increased the proportion of Bacteroidetes while reducing the Firmicutes/Bacteroidetes ratio.
Antipruritic effects of the probiotic strain LKM512 in adults with atopic dermatitis	The populations of *Atopobium* and *Bifidobacterium animalis* subsp. *lactis* in the probiotic group were significantly higher than those in the placebo group.
Effects of *Lactobacillus salivarius* LS01 (DSM 22775) treatment on adult atopic dermatitis: a randomized placebo-controlled study	The probiotic *Lactobacillus salivarius* significantly reduced serum levels of Th1 cytokines (IL-12 + IFN-gamma) and the Th1/Th2 ratio (IL-12 + IFN-gamma/IL-4 + IL-5). A statistically significant decrease in staphylococci was also observed in the feces of the probiotic-treated group.
The effect of probiotics on faecal microbiota and genotoxic activity of faecal water in patients with atopic dermatitis: a randomized, placebo-controlled study	Patients with AD exhibited higher fecal abundance of *C. perfringens* compared to healthy individuals. Probiotic supplementation significantly increased levels of *lactobacilli*, while the numbers of *Bifidobacteria* and *Bacteroidetes* remained unchanged in both healthy individuals and AD patients. No changes were observed regarding short-chain fatty acids.
LKM512 yogurt consumption improves the intestinal environment and induces the T-helper type 1 cytokine in adult patients with intractable AD	The intervention increased the abundance of *Bifidobacterium*, *Clostridium* cluster IV, and subcluster XIVa, as well as elevated levels of spermidine and butyrate.

## Supplementary Materials

The following supporting information can be downloaded at: https://www.mdpi.com/article/10.3390/jcm14010019/s1, Supplementary Material S1: Search strategy and Supplementary Material S2: Risk of Bias Assessments for Each Included Study.
