# Supplementary material for: Gut Dysbiosis and Adult Atopic Dermatitis: A Systematic Review
Source: J Clin Med. 2024 Dec 24;14(1):19. doi: 10.3390/jcm14010019 (PMC11721037; doi:10.3390/jcm14010019)
Supplement: Supplementary file 1 [file jcm-14-00019-s001.zip › jcm-3390205-supplementary S1.pdf]

The search conducted in PubMed utilized the following query and its corresponding translations adapted to each search engine: ("dermatitis, atopic"[MeSH Terms] AND "Gastrointestinal Microbiome"[MeSH Terms]) OR (("atopic dermatitis"[Title/Abstract] OR "atopic eczema"[Title/Abstract] OR "eczema"[Title/Abstract]) AND ("fecal microbiota"[Title/Abstract] OR "fecal microbiome"[Title/Abstract] OR "gut microbiota"[Title/Abstract] OR "gut microbiome"[Title/Abstract] OR "intestinal microbiota"[Title/Abstract] OR "intestinal microbiome"[Title/Abstract])). An initial search yielded 412 results.

In EMBASE, the search terms were ('dermatitis, atopic'/exp AND 'Gastrointestinal Microbiome'/exp) OR (('atopic dermatitis':ti,ab OR 'atopic eczema':ti,ab OR eczema:ti,ab) AND ('fecal microbiota':ti,ab OR 'fecal microbiome':ti,ab OR 'gut microbiota':ti,ab OR 'gut microbiome':ti,ab OR 'intestinal microbiota':ti,ab OR 'intestinal microbiome':ti,ab)).The initial search produced 1018 results.

In Web of Science, the search terms were ("dermatitis, atopic" AND "Gastrointestinal Microbiome") OR (("atopic dermatitis" OR "atopic eczema" OR eczema) AND ("fecal microbiota" OR "fecal microbiome" OR "gut microbiota" OR "gut microbiome" OR "intestinal microbiota" OR "intestinal microbiome")), yielding 1014 results.

The initial search yielded a total of 421 results in PubMed, 1018 in EMBASE, and 1014 in Web of Science, amounting to 2444 results. No filters were applied in any of the platforms. Duplicate articles were identified and excluded, reducing the count by 932 articles. The total number of articles for screening was therefore 1512. Following title screening, 377 articles were included for abstract review, with 1135 articles excluded for various reasons (multiple reasons could apply). Of these exclusions, 622 were for background or context, 564 for incorrect population, 149 for publication type, 20 for inadequate design, 3 for language, and 2 for incorrect outcome variable.

After abstract review (377 articles), 62 articles were selected for full-text review, with a total of 1449 articles excluded at this stage. The reasons for exclusion could also be multifactorial. Ultimately, following full-text review, a total of 15 articles were included in the systematic review.

The review was registered in Rayyan. Named: “Dermatitis atópica y microbioma en el adulto”
