# Supplementary material for: Gut Dysbiosis and Adult Atopic Dermatitis: A Systematic Review
Source: J Clin Med. 2024 Dec 24;14(1):19. doi: 10.3390/jcm14010019 (PMC11721037; doi:10.3390/jcm14010019)
Supplement: Supplementary file 1 [file jcm-14-00019-s001.zip › jcm-3390205-supplementary S2.pdf]

Risk of Bias Assessments for Each Included Study

Matsumoto et al.

- Design: Case-control study
- Case Selection: Low risk
- Control Selection: High risk
- Exposure Measurement: Low risk
- Confounding: High risk
- Outcome Attribution: Low risk
- Information Bias: Moderate risk

Hua et al.

- Design: Retrospective observational
- Participant Selection: Low risk
- Exposure Measurement: Low risk
- Confounding: High risk
- Information Bias: Low risk
- Outcome Attribution: Low risk
- Publication Bias: High risk

Li et al.

- Design: Case-control study
- Case Selection: Low risk
- Control Selection: High risk
- Exposure Measurement: Low risk
- Confounding: High risk
- Outcome Attribution: Low risk
- Information Bias: Low risk

Su et al.

- Participant Selection: Low risk
- Exposure Measurement: Low risk
- Confounding: High risk
- Information Bias: Low risk
- Outcome Attribution: Low risk
- Loss to Follow-up: Low risk

Liu et al.

- Design: Case-control study
- Case Selection: Moderate risk

- Control Selection: High risk
- Exposure Measurement: Low risk
- Confounding: High risk
- Outcome Attribution: Low risk
- Information Bias: Low risk

Han et al.

- Design: Case-control study
- Case Selection: High risk
- Control Selection: High risk
- Exposure Measurement: Low risk
- Confounding: High risk
- Outcome Attribution: Low risk
- Information Bias: Low risk

Fang et al.

- Design: Randomized Clinical Trial
- Randomization Sequence Generation: Low risk
- Allocation Concealment: Not specified
- Blinding of Participants and Personnel: Not specified
- Blinding of Outcome Assessment: Low risk
- Incomplete Outcome Data: Low risk
- Reporting Bias: Low risk
- Other Bias: High risk

Thirion et al.

- Design: Open-label Trial
- Randomization Sequence Generation: High risk
- Allocation Concealment: High risk
- Blinding of Participants and Personnel: High risk
- Blinding of Outcome Assessment: High risk
- Incomplete Outcome Data: Low risk
- Reporting Bias: Low risk
- Other Bias: High risk

Wang

- Design: Open-label Trial
- Randomization Sequence Generation: High risk
- Allocation Concealment: High risk
- Blinding of Participants and Personnel: High risk
- Blinding of Outcome Assessment: High risk

- Incomplete Outcome Data: Low risk
- Reporting Bias: Low risk
- Other Bias: High risk

Mashiah

- Design: Crossover Clinical Trial
- Randomization Sequence Generation: Low risk
- Allocation Concealment: Low risk
- Blinding of Participants and Personnel: Moderate risk
- Blinding of Outcome Assessment: High risk
- Incomplete Outcome Data: High risk
- Reporting Bias: Low risk
- Other Bias: High risk

Fang et al.

- Design: Clinical Trial
- Randomization Sequence Generation: Low risk
- Allocation Concealment: Not specified
- Blinding of Participants and Personnel: Not specified
- Blinding of Outcome Assessment: Not specified
- Incomplete Outcome Data: Moderate risk
- Reporting Bias: Low risk
- Other Bias: High risk

Matsumoto et al.

- Design: Clinical Trial
- Randomization Sequence Generation: Low risk
- Allocation Concealment: Low risk
- Blinding of Participants and Personnel: Low risk
- Blinding of Outcome Assessment: Low risk
- Incomplete Outcome Data: High risk
- Reporting Bias: Low risk
- Other Bias: High risk

Drago et al.

- Design: Clinical Trial
- Randomization Sequence Generation: Low risk
- Allocation Concealment: Low risk
- Blinding of Participants and Personnel: Low risk
- Blinding of Outcome Assessment: Low risk
- Incomplete Outcome Data: Low risk

- Reporting Bias: Low risk
- Other Bias: High risk

Roessler et al.

- Design: Crossover Clinical Trial
- Randomization Sequence Generation: Low risk
- Allocation Concealment: Low risk
- Blinding of Participants and Personnel: Low risk
- Blinding of Outcome Assessment: Low risk
- Incomplete Outcome Data: Low risk
- Reporting Bias: Low risk
- Other Bias: High risk

Matsumoto et al.

- Design: Crossover Clinical Trial
- Randomization Sequence Generation: Low risk
- Allocation Concealment: Low risk
- Blinding of Participants and Personnel: Low risk
- Blinding of Outcome Assessment: Low risk
- Incomplete Outcome Data: Low risk
- Reporting Bias: Low risk
- Other Bias: High risk
